# Establishment of Novel Murine Model showing Vascular Inflammation-derived Cognitive Dysfunction

**DOI:** 10.1038/s41598-019-40726-z

**Published:** 2019-03-11

**Authors:** Tsuyoshi Hashizume, Bo-Kyung Son, Sakiko Taniguchi, Koichi Ito, Yoshihiro Noda, Tamao Endo, Michiko Nanao-Hamai, Sumito Ogawa, Masahiro Akishita

**Affiliations:** 1 Department of Geriatric Medicine, Graduate School of Medicine, The University of Tokyo, Tokyo, Japan; 2 Institute of Gerontology, The University of Tokyo, Tokyo, Japan; 3 Department of Food and Physiological Models, Graduate School of Agricultural and Life Sciences, The University of Tokyo, Tokyo, Japan; 4 Animal Facility, Tokyo Metropolitan Institute of Gerontology, Tokyo, Japan

## Abstract

Inflammation is a critical feature of aging and its related diseases, including cardiovascular diseases. Recent epidemiological studies demonstrated that abdominal aortic aneurysm (AAA), an aging-related vascular pathological condition, is associated with cognitive decline. However, the underlying mechanism, especially the role of vascular inflammation, is largely unknown because of lack of an available animal model. In this study, we examined whether vascular inflammation affects synaptic and cognitive dysfunction, using an AAA mouse model. In young (3 months) and middle-aged (12 months) C57BL/6J mice, AAA was induced by angiotensin II infusion with calcium chloride application. After 4 weeks of induction, aortic diameter was significantly increased and excessive Mac3-positive inflammatory cells infiltrated the destroyed aorta in middle-aged mice. AAA-induced middle-aged mice further exhibited cognitive impairment. Neuronal loss was observed in the CA3 region of the hippocampus. IBA1/MHCII-double-positive microglia activation was also seen in the hippocampus, suggesting that vascular inflammation drives neuroinflammation and subsequent cognitive dysfunction. Furthermore, we found that senescence-accelerated mice prone 8 exhibited robust AAA formation and a marked decrease of cognitive and synaptic function in the hippocampus mediated by inflammation. In conclusion, this novel murine model convincingly suggested the occurrence of vascular inflammation-derived cognitive dysfunction.

## Introduction

The prevalence of abdominal aortic aneurysm (AAA), which is characterized by localized dilation of the abdominal aorta, increases by 40% every 5 years in men over 65 years old^1^, representing an aging-related vascular pathological condition with significant morbidity and mortality^1–5^. Inflammation is a critical feature of aging and its related diseases including AAA^6–9^. Infiltrated/residential immune cells play important roles through their release of inflammatory cytokines and proteases that mediate tissue degradation and dysfunction^10^. In the vessel, vascular aging and diseases are also attributed to inflammation^11,12^. Infiltration of circulating monocytes and local proliferation of macrophages in aged or diseased vessels have been observed in humans and animal models^13–15^, suggesting their central roles in vascular inflammation. Secretion of proinflammatory cytokines such as TNFα^16^ and IL-18^17^ and the production of chemokines by macrophages promote further recruitment of immune cells. Macrophages in atherosclerotic lesions are also known to have strong proteolytic activity and are thus considered to substantially participate in plaque destabilization and rupture^18^.

Emerging evidence indicates that aging-related vascular pathology is clinically associated with cognitive decline. For example, arterial stiffness, as assessed by high carotid-femoral pulse wave velocity, is related to cognitive impairment^19,20^. Atherosclerotic calcification is also associated with cognitive dysfunction^21^. Moreover, it is reported that exposure to vascular risk factors, such as hypertension, diabetes, smoking and obesity, in midlife accelerates cognitive decline^22^. In aging-related cognitive decline, recent studies revealed that inflammation in the hippocampus also plays a critical role, and microglia are particularly central to mediating its effects^23–26^. Although inflammation has been suggested to contribute to this relationship between aging-related vascular pathology and cognitive decline^27^, the effects of vascular inflammation on cognitive function have not been fully determined, due to lack of available animal models.

We recently established an AAA model by local calcium chloride (CaCl_2_) application and continuous angiotensin II (AngII) infusion, and found in this model that vascular inflammation is a pathological hallmark caused by macrophage infiltration and increased secretion or expression of inflammatory cytokines^28^. Interestingly, a recent epidemiologic study demonstrated that AAA is related to cognitive impairment. AAA patients showed cognitive impairment in the domains of immediate and delayed memory, and cognitive dysfunction was best predicted by increasing aortic diameter, which was positively related to C-reactive protein^29^. Given that vascular inflammation has the potential to drive systemic inflammation, using this model, we might examine the effects of vascular inflammation on neuroinflammation through disturbance of systemic control.

In this study, to address this issue, we examined the influence of vascular inflammation on cognitive impairment, using three experimental approaches: (1) a natural murine aging model; (2) AAA induction in a natural murine model of aging; (3) AAA induction in a murine model of accelerated aging. After 4 weeks of AAA induction, increased diameter of the abdominal aorta was observed in 12-month-old (middle-aged, M) mice compared with 3-month-old (young, Y) mice. Intriguingly, AAA-induced M mice exhibited a decrease of cognitive function, which was also seen in 24-month-old (aged, A) mice. Likewise, AAA induction accelerated cognitive aging in senescence-accelerated mice prone 8 (SAMP8). Mechanistically, infiltration of Mac3-positive inflammatory cells was seen in the aorta and IBA1/MHCII-double-positive microglia activation in the hippocampus of AAA-induced M mice, suggesting that vascular inflammation drives neuroinflammation and subsequent cognitive impairment. Thus, this novel murine model convincingly suggested the occurrence of vascular inflammation-derived cognitive dysfunction.

## Results

### Aged mice exhibited significant decline of spatial memory and synaptic dysfunction

**Figure 1:**
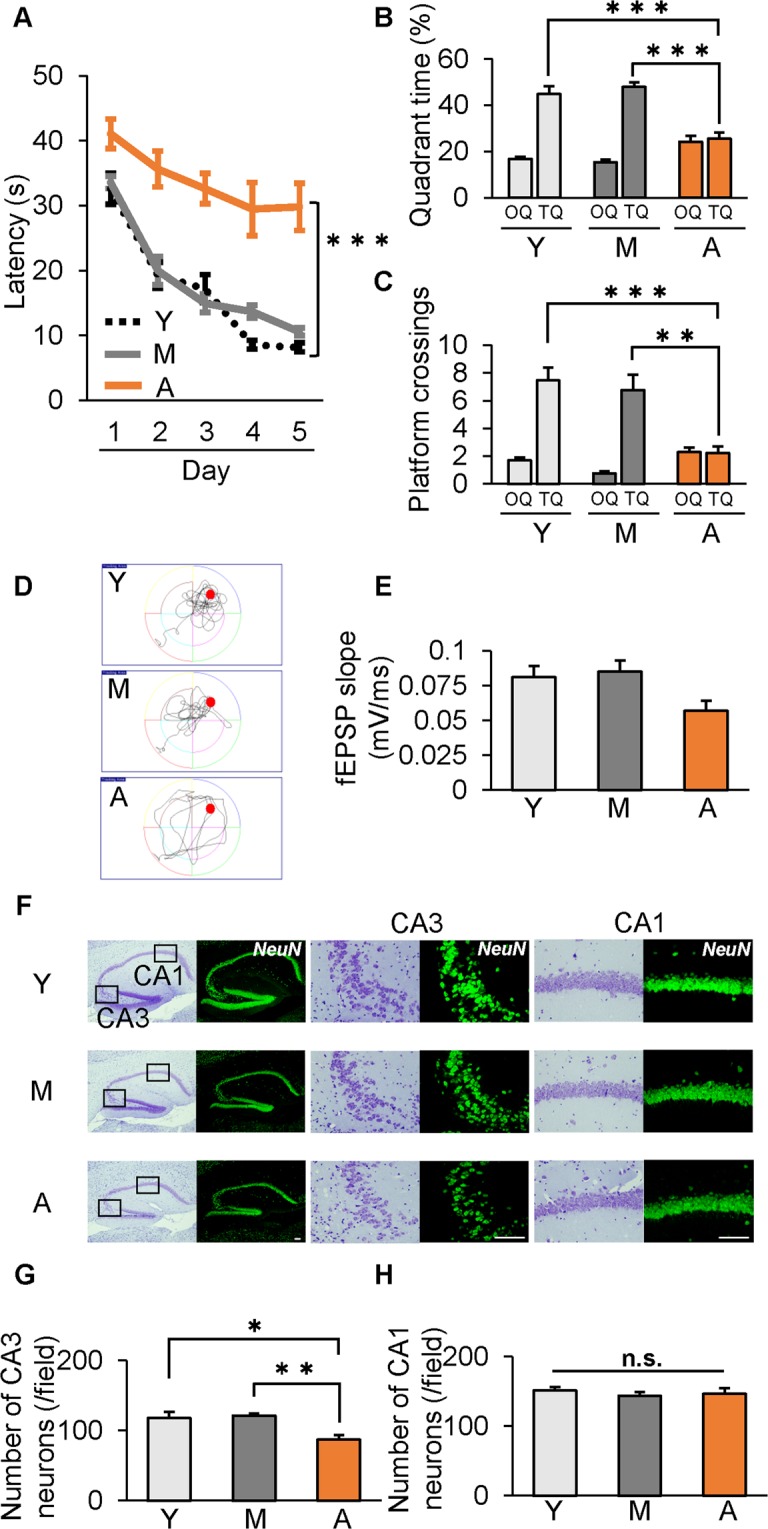
Age-dependent decline of neuronal cells and cognitive function in wild-type mice. (**A**–**D**) Morris water maze test in C57BL/6J mice aged 3 months (young, Y), 12 months (middle-aged, M), and 24 months (aged, A) old. (**A**) “A” mice showed significant impairment of escape latency in the hidden platform trial. (**B**,**C**) Swim time spent (**B**) and number of platform crossings (**C**) in the target quadrant (TQ) vs. other quadrants (OQs). Values of the three OQs were combined and averaged. (**D**) Representative searching strategy in the probe test. (**E**) Slope of fEPSP significantly decreased in “A” mice. (**F**) Representative low- and high-power photomicrographs of sections of hippocampal CA3 and CA1 regions with Nissl and NeuN staining. (**G**,**H**) Quantification of hippocampal neuronal populations in CA3(G) and CA1(H) regions. *p < 0.05, **p < 0.01, ***p < 0.001. Scale bar = 100 µm. n = 13 for Y mice, n = 13 for M mice and n = 9 for A mice (**A**–**D**); n = 19 for Y mice, n = 19 for M mice and n = 9 for A mice (**E**); n = 5 mice/group (**F**–**H**).

We first investigated cognitive function in normal aging in Y, M and A mice (n = 9–13 mice per group), by examination of spatial learning by the Morris water maze test, synaptic strength (slope of fEPSP) and neuron number in the hippocampus. “A” mice performed significantly worse in the hidden platform trial (p < 0.001; Fig. 1A), spent significantly less time in the target quadrant (TQ) in the probe test (Fig. 1B,D), and crossed the targeted area less frequently (Fig. 1C,D), compared with Y and M mice. The slope of fEPSP decreased, but not statistically significantly (0.085 to 0.057, P = 0.111), in “A” mice (Fig. 1E). We then analyzed the population of neuronal cells in the hippocampus by Nissl and NeuN staining. The number of neurons in the CA3 region of the hippocampus significantly decreased in “A” mice (p < 0.05; Fig. 1F,G). However, the number of neurons in CA1 did not change with age (Fig. 1F,H). We found only in “A” mice, a decline of spatial memory and synaptic strength, and loss of neuronal cells in the hippocampus.

### Increase in inflammatory cells in aged aorta and hippocampus

**Figure 2:**
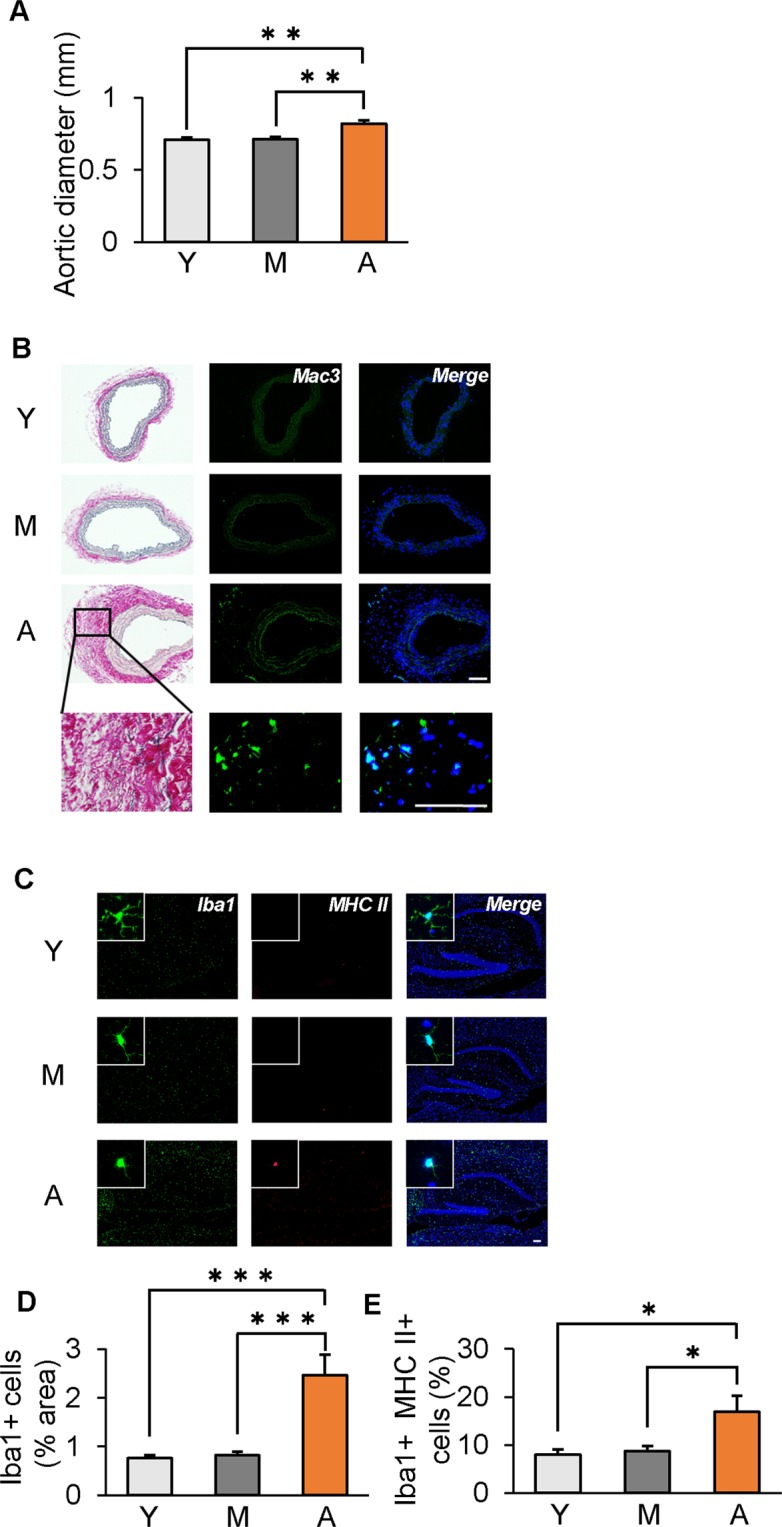
Age-dependent increase in inflammatory cells in both aorta and hippocampus in wild-type mice. (**A**) Quantification of infrarenal aortic diameter at 3 months (young, Y), 12 months (middle-aged, M), and 24 months (aged, A) old. (**B**) Immunofluorescent staining for macrophages (green: Mac3, blue: DAPI) and EVG staining. (**C**) Micrographs depict immunofluorescent labeling for IBA1 and MHCII (green: Iba1, red: MHCII, blue: DAPI). (**D**) Number of cells expressing IBA1, a marker of microglia and macrophages, increased in A mice. (**E**) The proportion of IBA1-positive cells expressing MHCII was also greater in the hippocampus of A mice. *p < 0.05, **p < 0.01, ***p < 0.001. Scale bar = 100 µm. n = 17 for Y mice, n = 19 for M mice and n = 11 for A mice (**A**,**B**); n = 5 mice/group (**C**–**E**).

Because inflammation is a common feature of aging tissues, and immune cells play central roles, we examined the existence of inflammatory cells in the aorta and hippocampus of Y, M and A mice (n = 11–19 mice per group), using histological analysis. Compared with those of Y and M mice, the abdominal aorta of A mice showed increased diameter (p < 0.01) and fibrotic tissue deposition by EVG staining, and Mac3-positive immune cells were also seen (Fig. 2A,B). Similarly, the proportion of not only IBA1-positive cells (microglia), but also IBA1/MHCII-double-positive cells (activated microglia) was greater in the hippocampus of A mice (p < 0.05; Fig. 2C–E). These findings suggest that macrophages and activated microglia exist in the aged aorta and hippocampus, respectively, suggesting their possible roles in age-related inflammation even in a steady state.

### Cognitive decline was accelerated by vascular inflammation in M Mice

**Figure 3:**
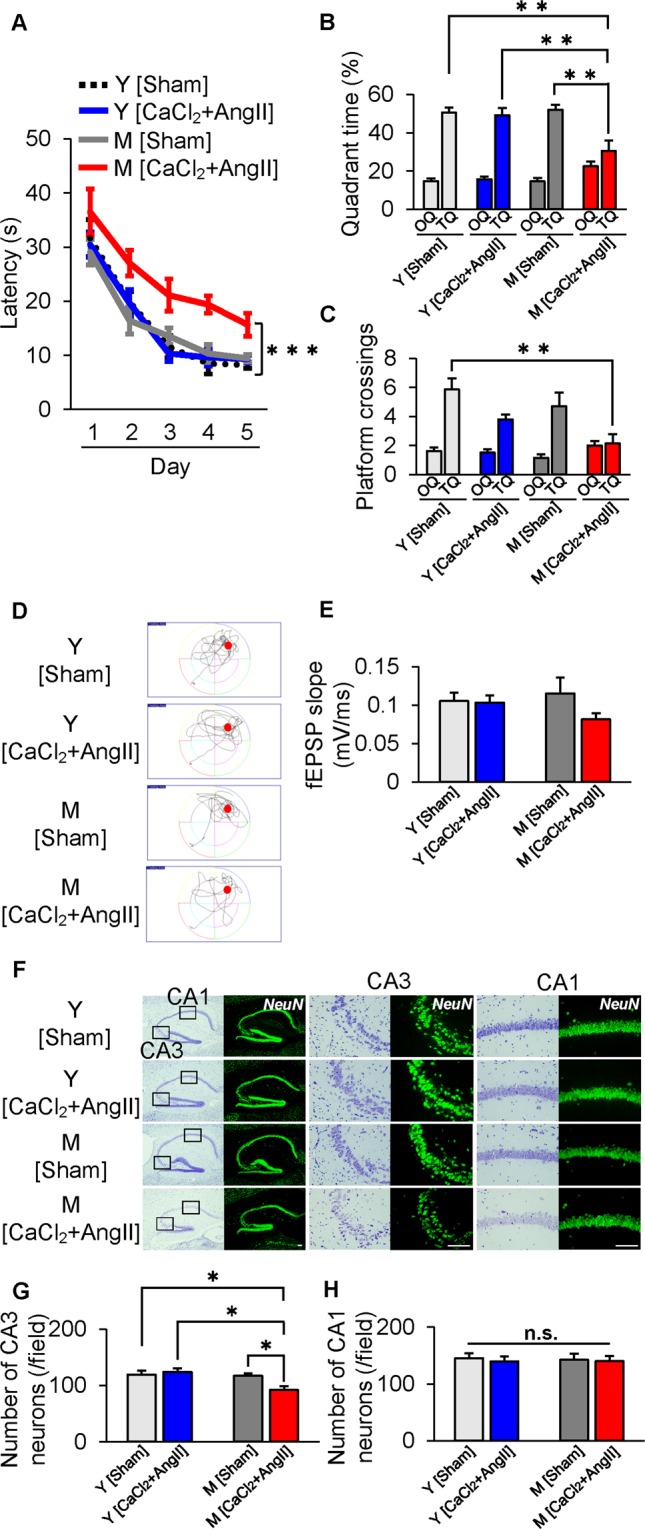
Vascular inflammation induced by abdominal aortic aneurysm accelerates synaptic and cognitive dysfunction in middle-aged mice. (**A**–**D**) Morris water maze test in 3-month- (young, Y) and 12-month- (middle-aged, M) old mice at 4 weeks after sham operation (Sham) or angiotensin II (AngII) infusion with calcium chloride (CaCl_2_) application (CaCl_2_ + AngII). (**A**) M mice after CaCl_2_ + AngII showed significant impairment of escape latency in the hidden platform trial. (**B**,**C**) Swim time spent (**B**) and number of platform crossings (**C**) in target quadrant (TQ) vs. other quadrants (OQs). Values of the three OQs were combined and averaged. (**D**) Representative searching strategy in the probe test. (**E**) Slope of fEPSP decreased in M mice after CaCl_2_ + AngII. (**F**) Representative low- and high-power photomicrographs of sections of hippocampal CA3 and CA1 regions with Nissl and NeuN staining. (**G**,**H**) Quantification of hippocampal neuronal populations in CA3(G) and CA1(H) regions. *p < 0.05, **p < 0.01, ***p < 0.001. Scale bar = 100 µm. n = 7 for Y [Sham], n = 10 for Y [CaCl_2_ + AngII], n = 7 for M [Sham] and n = 7 for M [CaCl_2_ + AngII] (**A**–**D**); n = 16 for Y [Sham], n = 14 for Y [CaCl_2_ + AngII], n = 9 for M [Sham] and n = 10 for M [CaCl_2_ + AngII] (**E**); n = 5 mice/group (**F**–**H**).

Next, to test the effect of vascular inflammation on cognitive function, we used an AAA mouse model (n = 7–10 mice per group). When AAA was induced in M mice, latent time in the hidden platform trial significantly increased, compared with that in both AAA-induced Y mice and sham-operated M mice (p < 0.001; Fig. 3A). Similarly, AAA-induced M mice spent significantly less time in TQ in the probe test (Fig. 3B,D), and crossed the targeted area less frequently (Fig. 3C,D). For synaptic strength, the slope of fEPSP decreased from 0.115 ± 0.020 to 0.081 ± 0.008 in AAA-induced M mice, but not statistically significantly (P = 0.273; Fig. 3E). The number of neurons in the CA3 region of the hippocampus significantly decreased in AAA-induced mice (p < 0.05; Fig. 3F,G), whereas AAA did not affect the number of neurons in the CA1 region (Fig. 3F,H).

### Vascular inflammation induces neuroinflammation as well as AAA formation in M Mice

**Figure 4:**
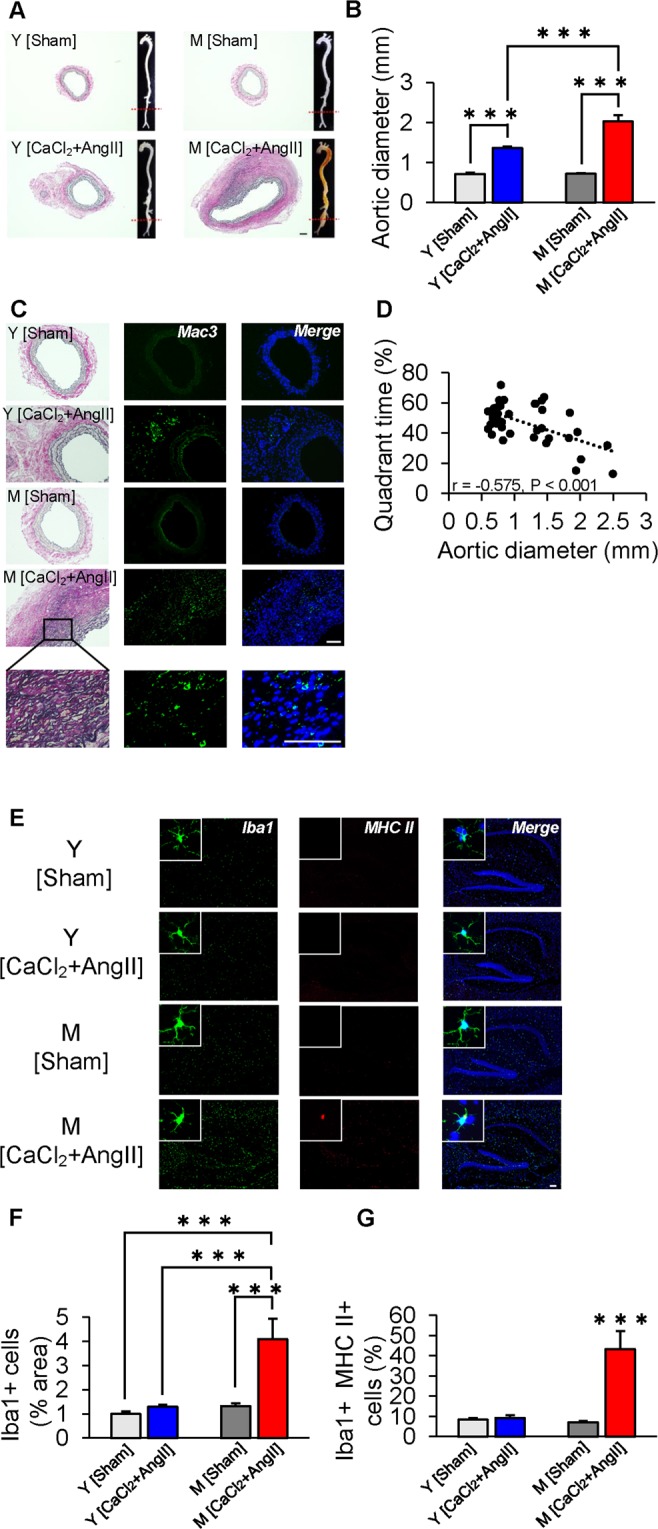
Enhanced aortic infiltration of macrophages and microglial activation in hippocampus of middle-aged mice induced by abdominal aortic aneurysm (AAA). (**A**) Representative aorta and histopathological analysis of infrarenal aorta in 3-month- (young, Y) and 12-month- (middle-aged, M) old mice after sham operation (Sham) or angiotensin II (AngII) infusion with CaCl_2_ application (CaCl_2_ + AngII). (**B**) Quantification of infrarenal aortic diameter. (**C**) Immunofluorescent staining for macrophages (green: Mac3, blue: DAPI) and EVG staining. (**D**) There was an inverse correlation between infrarenal aortic diameter and quadrant time in the probe trial. (**E**) Micrographs depict immunofluorescent labeling for IBA1 and MHCII (green: Iba1, red: MHCII, blue: DAPI). (**F**) Number of cells expressing IBA1, a marker of microglia and macrophages, was increased in M mice by AAA induction. (**G**) The proportion of IBA1-positive cells expressing MHCII was also greater in the hippocampus of AAA-induced M mice. ***p < 0.001. Scale bar = 100 µm. n = 7 for Y [Sham], n = 10 for Y [CaCl_2_ + AngII], n = 7 for M [Sham] and n = 7 for M [CaCl_2_ + AngII] (**A**–**D**); n = 5 mice/group (**E**–**G**).

We further investigated the role of vascular inflammation in neuroinflammation from a mechanistic view. Increased diameter of the infrarenal aorta was observed in M mice after AAA induction, compared with Y mice after AAA induction (n = 7–10 mice per group, p < 0.001; Fig. 4A,B). On histological analysis, elastic fiber destruction and fibrotic tissue deposition were observed by EVG staining, and Mac3-positive cells were seen in the destroyed area, implying that infiltration of immune cells into the aorta was associated with inflammatory responses and vascular remodeling (Fig. 4A,C). Furthermore, in the hippocampus of AAA-induced M mice, the proportion of IBA1/MHCII-double-positive cells as well as IBA1-positive cells was greater (p < 0.05; Fig. 4E–G). We also found an inverse correlation between infrarenal aortic diameter and quadrant time in the probe trial (r = −0.575, p < 0.001; Fig. 4D), supporting that vascular inflammation leads to cognitive dysfunction through neuronal inflammation.

### Vascular inflammation accelerates cognitive decline in SAMP8

**Figure 5:**
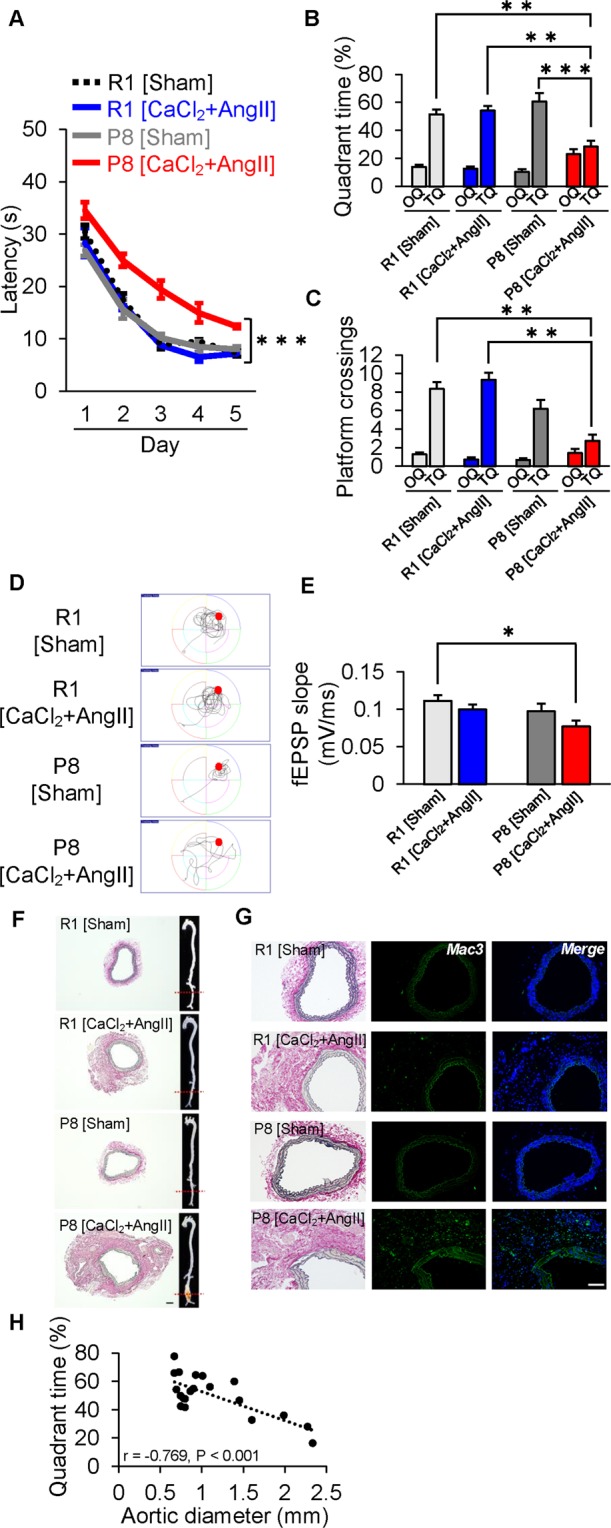
Vascular inflammation induced by abdominal aortic aneurysm accelerates cognitive dysfunction in senescence-accelerated mice prone 8 (SAMP8). (**A**–**D**) Morris water maze test in senescence-accelerated mice resistant 1 (SAMR1) and SAMP8 4 weeks after sham operation (Sham) or angiotensin II (AngII) infusion with calcium chloride (CaCl_2_) application (CaCl_2_ + AngII). (**A**) SAMP8 after CaCl_2_ + AngII showed significant impairment of escape latency in the hidden platform trial. (**B**,**C**) Swim time spent (**B**) and number of platform crossings (**C**) in target quadrant (TQ) vs. other quadrants (OQs). Values of the three OQs were combined and averaged. (**D**) Representative searching strategy in the probe test. (**E**) Slope of fEPSP significantly decreased in SAMP8 after CaCl_2_ + AngII. (**F**) Representative aorta and histopathological analysis of infrarenal aorta in SAMR1 and SAMP8 after sham operation (Sham) or AngII infusion with CaCl_2_ application (CaCl_2_ + AngII). (**G**) Immunofluorescent staining for macrophages (green: Mac3, blue: DAPI) and EVG staining. (**H**) There was an inverse correlation between infrarenal aortic diameter and quadrant time in the probe trial. *p < 0.05, **p < 0.01, ***p < 0.001. Scale bar = 100 µm. n = 6 for SAMR1 [Sham], n = 5 for SAMR1 [CaCl_2_ + AngII], n = 6 for SAMP8 [Sham] and n = 4 for SAMP8 [CaCl_2_ + AngII] (**A**–**D**); n = 15 for SAMR1 [Sham], n = 12 for SAMR1 [CaCl_2_ + AngII], n = 13 for SAMP8 [Sham] and n = 10 for SAMP8 [CaCl_2_ + AngII] (**E**).

Finally, using SAMP8, whose typical phenotype is characterized by cognitive decline, we confirmed the impact of vascular inflammation on cognitive aging. AAA-induced SAMP8 performed significantly worse in the hidden platform trial (n = 4–6 mice per group, p < 0.001; Fig. 5A), spent significantly less time in TQ in the probe test (Fig. 5B,D), and crossed the targeted area less frequently (Fig. 5C,D), compared with AAA-induced SAMR1 and sham-operated SAMP8 mice. Also, the slope of fEPSP significantly decreased in AAA-induced SAMP8 (Fig. 5E). Increased diameter of the infrarenal aorta, elastic fiber destruction and fibrotic tissue deposition were observed in SAMP8 with AAA induction, and especially, Mac3-positive cells were seen in the destroyed area (Fig. 5F,G). In AAA-induced SAMP8, we found a similar correlation between infrarenal aortic diameter and quadrant time in the probe trial, as seen in AAA-induced M mice (r = −0.769, p < 0.001; Fig. 5H). Thus, we could confirm that vascular inflammation accelerates cognitive decline in SAMP8.

## Discussion

The present study investigated whether vascular inflammation drives synaptic dysfunction and cognitive decline through neuroinflammation, using a newly established mouse model. In AAA-induced M mice, loss of neuronal cells and decrease of cognitive function were seen. Synaptic function was decreased in AAA-induced M mice, but not statistically significantly. We also confirmed similar observations in AAA-induced SAMP8 mice. Thus, using an AAA model, we found that vascular inflammation drives cognitive dysfunction, and further found, mechanistically, that macrophage-mediated inflammation in the aorta leads to microglial activation in the hippocampus, which is likely to be the basis of synaptic and cognitive impairment.

Previous studies have focused on the role of neuroinflammation as a mechanism of cognitive dysfunction^23–26^. In the present study, we examined the remote effects of vascular inflammation on neuroinflammation, followed by cognitive dysfunction. Since vascular inflammation is a pathological hallmark of AAA, we used an AAA mouse model. We found that M mice with AAA induction exhibit neuroinflammation, which is accountable for cognitive dysfunction. This inflammatory interaction of the vascular-neural axis suggests that some circulating factors might be released from the aorta, affect systemic inflammation, and then with involvement of humoral factors or participation of other immune cells in the hippocampus, proceed to induce neuroinflammation and cognitive impairment. Previous studies support this hypothesis. For example, AAA has been suggested to serve as a source of circulating inflammatory cytokines^30^. Previous examination of the circulating plasma profile of AAA subjects demonstrated that the plasma inflammatory cytokine score calculated from eotaxin, RANTES, and high sensitivity C-reactive protein suggested a strong risk association with AAA, independent of age, sex, history of ischemic heart disease, and smoking^31^. Furthermore, macrophage content in the inflamed vessel wall is directly correlated with circulating monocyte level^32^, suggesting that vascular inflammation has sufficient potential to lead to systemic inflammation. Proinflammatory cytokines, such as IL-1, IL-6 and TNFα, are also known to have a strong association with cognitive decline in humans and animal models^33–36^. Some studies have found that the IL-6-triggered positive feedback loop for NFkB signaling (or the IL-6 amplifier/inflammation amplifier) is critical to the development of chronic inflammation^37^, and that spinal cord endothelial cell stimulation can trigger the IL-6 amplifier to cause T cell migration into the central nervous system^38^. Our experiments indicate that vascular inflammation might activate inflammation amplifier in spinal cord endothelial cells through systemic inflammation and induce neuroinflammation. However, identification of the humoral factors originating from AAA contributing to neuroinflammation will need further investigation.

Neuroinflammation has been suggested to contribute to neuronal loss and synaptic dysfunction, and microglia/macrophages are predominant cellular sources mediating these effects^23–26^. Microglial activation leads to the release of pro-inflammatory cytokines, which contribute to neuronal loss^39,40^. Consistent with previous studies, we demonstrated that in AAA-induced M mice, the number of neurons in the CA3 region of the hippocampus significantly decreased, and IBA1/MHCII-double-positive cells, which represented active microglia and macrophages, increased in the hippocampus (we have no data on this point in SAMP8). It is possible that neuronal loss in the CA3 region reflects ‘frailty,’ an aging-related state of vulnerability to poor resolution of homoeostasis after a stressor event^41^, on the grounds that CA3 neuronal viability is selectively affected by stress and immune challenge^42,43^. Interestingly, given that previous reports suggest that, under normal conditions, microglia are not a particularly robust source of MHCII, and antigen-presenting cells in the brain are a mixed population of perivascular macrophages that have penetrated the blood-brain barrier (BBB)^44^, AAA might affect the cellular source of antigen-presenting cells in the hippocampus. Previous studies further support this notion of an essential role of perivascular macrophages in the initiation of proinflammatory cascades in the brain after infection^45,46^. In addition, AAA can induce cerebrovascular inflammation, which is reported to cause cognitive dysfunction^47,48^. Further examination will be needed to confirm the effects of AAA on the characteristics of circulating immune cells, BBB breakdown and cerebrovascular inflammation.

It was reported that the same level of hypertension was induced in both young (3 months) and aged (24 months) C57BL/6 mice by chronic infusion of AngII, but only in aged AngII-induced hypertensive mice were cerebral blood flow autoregulation and BBB markedly disrupted^49^. Also in our study, there was no difference in blood pressure between Y mice and M mice after CaCl_2 + _AngII (Supplementary Fig. 1), and only M mice with a combination of CaCl_2_ application and AngII infusion exhibited markedly increased diameter of the abdominal aorta and a significant decrease of cognitive function. Although the BBB is impermeable to AngII, local brain AngII has possible physiological and pharmacological functions in the neuronal system^50^. In addition, AngII might directly affect cognitive function through BBB disruption. However, considering that M mice after AngII infusion without CaCl_2_ application did not show a decrease of cognitive function (Supplementary Fig. 2), we suppose that cognitive dysfunction of M mice after CaCl_2 + _AngII was not caused by the direct effect of AngII. Although some previous studies used AngII infusion alone as an intervention to induce AAA, the limitation of this procedure for mechanistic investigations including inflammation was low reproducibility. It is noteworthy that the present model could induce vascular inflammation and AAA with high reproducibility.

In conclusion, our study demonstrates that AAA, induced by AngII infusion with CaCl_2_ application, accelerates neuroinflammation followed by cognitive dysfunction in 12-month-old wild type C57BL/6J mice and 4-month-old SAMP8, like those observed in 24-month-old wild type mice. This novel model suggested the occurrence of vascular inflammation-derived cognitive dysfunction. Further investigation of humoral factors originating from vascular inflammation, accelerating neuroinflammation, could suggest novel potential targets for diagnostic and therapeutic exploitation as well as a diagnostic biomarker.

## Methods

### Animals

We conducted a three-step experiment: (1) a natural murine aging model; (2) AAA induction in a natural murine model of aging; (3) AAA induction in a murine model of accelerated aging. Each group was independently processed in each experiment. C57BL/6J male mice aged 3 months (young, Y), 12 months (middle-aged, M), and 24 months (aged, A) old were obtained from the Tokyo Metropolitan Institute of Gerontology. Male SAMP8 and control senescence-accelerated mice resistant 1 (SAMR1) were purchased from Japan SLC, Inc. (Shizuoka, Japan). Mice were all housed in a room at 22 ± 2 °C with an automatic light cycle (12 h light/12 h dark) and relative humidity of 40–60%, and given free access to water and standard laboratory chow. All experimental protocols were approved by the Ethics Committee for Animal Experimentation of the Graduate School of Medicine, the University of Tokyo, and conducted in accordance with the Guidelines for the Care and Use of Laboratory Animals of the Department of Medicine, the University of Tokyo.

### Murine AAA model

To induce AAA, peri-aortic application of CaCl_2_ was performed on the abdominal aorta, followed by 4-week subcutaneous infusion of AngII (2,000 ng kg^−1^ min^−1^) via an implanted osmotic pump^51^. In detail, mice were anesthetized and underwent laparotomy. The abdominal aorta between the renal arteries and bifurcation of the iliac arteries was isolated from the surrounding retroperitoneal structures, and 0.5 M CaCl_2_ was applied to the external surface of the infrarenal aorta. NaCl (0.9%) was substituted for CaCl_2_ in sham control mice. The aorta was rinsed with 0.9% sterile saline after 15 min and the incision was closed.

### Morris water maze test

The training apparatus was a circular pool (120 cm in diameter) containing water at 22 °C ± 1 °C. In the testing room, large black cue symbols were put on each wall for spatial orientation. A platform (9 cm in diameter) was submerged 0.5 cm under the water surface. The water maze protocol was performed as previously described^52–54^ with slight modifications. Mice were tested with 3 days of visible platform trial, followed by 5 days of hidden platform trial (four trials per session per day). Mice were allowed to search for the platform for a maximum of 60 sec. If the mouse did not find the platform within 60 seconds, it was guided to it. Mice were then allowed to remain on the platform for 15 seconds. The exclusion criterion of 20 seconds to reach the platform for the last 4 trials of the visible platform task was used. The navigation of mice was recorded with a video camera, and the escape latency time to locate the hidden platform was recorded. The probe trial was performed at the end of the last session. During the probe trial, the platform was removed and mice were allowed to swim in the pool for 60 seconds. Swim time spent and the number of platform crossings in the target quadrant (TQ) vs. other quadrants (OQs) were recorded.

### Hippocampal slice physiology

Hippocampal slices were made as previously described^55^. Briefly, mice were decapitated and the brain was removed from the skull under ice-cold sucrose-rich slicing solution (SRSS) containing 85 NaCl, 2.5 KCl, 1.25 NaH_2_PO_4_, 25 NaHCO_3_, 25 glucose, 75 sucrose, 0.5 CaCl_2_, 4 MgCl_2_, and 0.6 ascorbic acid (in mM). Slices were made with a DSK-Linear Slicer Model Pro 10 (Dosaka, Kyoto, Japan) at 350 µm and incubated in SRSS. Then SRSS was replaced with artificial cerebrospinal fluid (ACSF) containing 125 NaCl, 2.4 KCl, 1.2 NaH_2_PO_4_, 25 NaHCO_3_, 25 glucose, 1 CaCl_2_, and 2 MgCl_2_ (in mM), at a speed of 1 mL/min. Both SRSS and ACSF were equilibrated with 95% O_2_/5% CO_2_. After 1 h, individual slices were transferred to a recording chamber and perfused continuously with ACSF containing 125 NaCl, 2.4 KCl, 1.2 NaH_2_PO_4_, 25 NaHCO_3_, 25 glucose, 2 CaCl_2_, and 1 MgCl_2_ (in mM) at a speed of 1–3 mL/min at room temperature. Glass electrodes were pulled from borosilicate glass and filled with 2 M NaCl for recording electrodes and ACSF for stimulation electrodes. Electrical stimuli were delivered by an electric stimulator SEN-3301 and isolator SS-202 J (Nihon Kohden, Tokyo, Japan) with Schaffer collateral stimulation, and evoked field EPSP (fEPSP) was recorded from recording electrodes positioned in the hippocampal stratum radiatum placed more than 100 µm from pyramidal cells in the CA1 region. Data were collected and analyzed by pClamp 10.2 (Axon Instruments, California, USA).

### Histological analysis

#### Aorta

Aortas were embedded in paraffin and then 5-μm-thick serial sections were prepared for elastic van Gieson (EVG) staining. Digital images of EVG-stained aortas with a reference scale were used for absolute measurement of diameter. Paraffin-embedded aortic sections were also used for immunohistochemical staining. After deparaffinization and blocking, serial sections were incubated with anti-Mac3 antibody (dilution 1:200; BD Pharmingen) for macrophages in mice, followed by biotinylated secondary antibodies (1:200; Dako). For detection, anti-streptavidin-conjugated AlexFluor 488 (1:200; Invitrogen) was used. The nuclei were stained with 4′, 6-diamidino-2-phenylindole (1:5,000; Sigma-Aldrich) after the final series of washes.

#### Hippocampus

Following perfusion with 4% paraformaldehyde (PFA) in PBS, brains were postfixed for 48 h in 4% PFA followed by cryoprotection with sucrose overnight at 4 °C. Brains were then infiltrated in a 1:1 solution of sucrose and cryo-protectant medium (OCT Tissue-Tek, USA), embedded in 100% OCT, and snap-frozen in liquid nitrogen. Frozen 10-μm-thick brain sections were used for Nissl staining, which was performed as previously described^56^. For immunohistochemical staining, brain sections were blocked and incubated with the following primary antibodies: anti-NeuN (1:200; Abcam), anti-Iba1 (1:200; Bioss), or anti-MHC II (1:200; BIO-RAD), followed by biotinylated secondary antibodies (1:200; Dako). For detection, anti-streptavidin-conjugated AlexFluor 488 or AlexFluor 594 (1:200; Invitrogen) was used. Then, 4′, 6-diamidino-2-phenylindole (1:5,000; Sigma-Aldrich) was used to reveal nuclei. Neuronal cells in the hippocampal CA3 and CA1 pyramidal cell layers were each counted in 4–5 sections (at 100 μm interval) in 5 animals, and averaged. Regions of interest were traced and overlaid with a counting grid for the optical dissector method. Neurons were counted within the set dissector area. Only intact neurons with a clearly defined cell body and nucleus were counted. The numbers of Iba1- and MHCII-positive neurons in the hippocampus were counted in 4′, 6-diamidino-2-phenylindole-positive cells using National Institutes of Health Image J Software. All counts were performed blind to the experimental condition.

### Statistical analyses

All data are presented as mean ± S.E.M. Morris water maze data were analyzed using two-way repeated measures analysis of variance, and other data were analyzed with one-way analysis of variance followed by Bonferroni post hoc test. All data were analyzed using Prism 6.0 (GraphPad Software). A p value of less than 0.05 was considered significant.

## Supplementary information

Supplementary information
