# Supplementary material for: Establishment of Novel Murine Model showing Vascular Inflammation-derived Cognitive Dysfunction
Source: Sci Rep. 2019 Mar 11;9:4023. doi: 10.1038/s41598-019-40726-z (PMC6411753; doi:10.1038/s41598-019-40726-z)
Supplement: Supplementary file 1 — Supplementary information [file 41598_2019_40726_MOESM1_ESM.pdf]

Establishment of Novel Murine Model showing Vascular Inflammation-derived Cognitive Dysfunction

- Supplementary information -

Tsuyoshi Hashizume, MD, PhD; Bo-Kyung Son, PhD; Sakiko Taniguchi, MD; Koichi Ito, MD, PhD; Yoshihiro Noda, MD, PhD; Tamao Endo, MD, PhD; Michiko Nanao-Hamai, MD, PhD; Sumito Ogawa, MD, PhD; Masahiro Akishita* MD, PhD

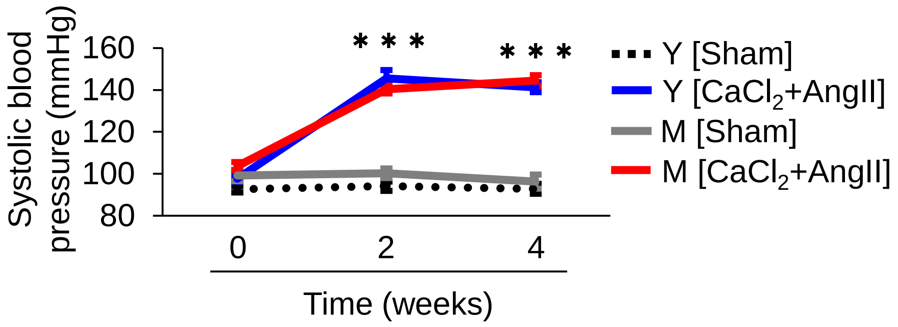

Supplementary Figure 1. Changes of systolic blood pressure after sham operation (Sham) or angiotensin II (AngII) infusion with calcium chloride ($CaCl_2$) application ($CaCl_2$ + AngII) in 3-month- (young, Y) and 12-month- (middle-aged, M) old mice. Systolic blood pressure was higher in AAA-induced Y and M mice than in sham groups, but there was no difference between AAA-induced Y and M mice. ***$p<0.001$. n=7 for Y [Sham], n=10 for Y [$CaCl_2$+AngII], n=7 for M [Sham] and n=7 for M [$CaCl_2$+AngII].

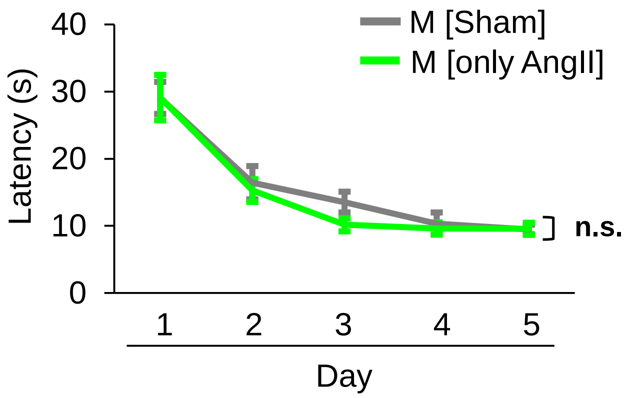

Supplementary Figure 2. Escape latency in hidden platform trial in 12-month- (middle-aged, M) old mice after angiotensin II (AngII) infusion without $CaCl_2$ application. There was no difference in escape latency in the hidden platform trial between M mice after sham operation (Sham) and M mice after AngII infusion without $CaCl_2$ application (only AngII). n=7 for M [Sham] and n=7 for M [only AngII].
